# Endolysins for precision antimicrobial therapy and rapid bacterial detection: translational challenges and future directions

**DOI:** 10.3389/fphar.2026.1943991

**Published:** 2026-10-29

**Authors:** Fazal Mehmood Khan, Mohammed Zourob

**Affiliations:** Department of Chemistry, College of Science and General Studies, Alfaisal University, Riyadh, Saudi Arabia

**Keywords:** antimicrobial resistance, bacteriophages, cell wall-binding domains, multidrug-resistant bacteria, phage-derived proteins, protein engineering, receptor-binding proteins

## Abstract

Antimicrobial resistance (AMR) represents a major global health challenge, necessitating the development of alternative therapeutic and diagnostic strategies. Among emerging approaches, bacteriophage-derived endolysins have attracted considerable attention as promising tools for combating bacterial infections. Endolysins are phage-encoded peptidoglycan-degrading enzymes that have shown significant potential for both antimicrobial and diagnostic applications against a wide range of bacterial pathogens. This review summarizes the current knowledge of endolysins, highlighting their rapid bacteriolytic activity, high target specificity, and diverse therapeutic and diagnostic applications. Beyond their antimicrobial potential, endolysin-derived cell wall-binding domains (CBDs) and phage receptor-binding proteins (RBPs) have emerged as highly specific recognition molecules for the rapid detection of bacterial pathogens in clinical diagnostics and food safety. Rapid and accurate pathogen detection is essential for timely disease management, infection control, and the prevention of foodborne contamination. Despite these promising attributes, several challenges continue to limit the clinical and industrial translation of endolysins, including constraints related to protein expression, stability, formulation, and large-scale production. Furthermore, the outer membrane of Gram-negative bacteria remains a major obstacle to endolysin activity by restricting access to the peptidoglycan layer. Recent advances in protein engineering, membrane-permeabilization strategies, and delivery approaches have provided promising solutions to overcome these limitations. Overall, this review provides a comprehensive overview of the current advances, translational challenges, and future prospects of engineered endolysins as precision antimicrobial agents and diagnostic tools for combating antimicrobial-resistant bacterial pathogens.

## 1 Introduction

### 1.1 The growing crisis of bacterial resistance

The rise of bacterial resistance to multiple antibiotics poses a significant threat to global public health (Woolhouse and Farrar, 2014). Antimicrobial resistance (AMR) is associated with substantial morbidity and mortality worldwide, leading the World Health Organization (WHO) to recognize AMR as a major global health challenge (Larsson and Flach, 2022). In 2019, AMR was estimated to contribute to approximately 3.57–4.95 million deaths globally (Murray et al., 2022). Furthermore, projections suggest that AMR-associated deaths may reach nearly 10 million annually by 2050 if effective intervention strategies are not implemented (Trotter et al., 2019). The emergence and spread of antibiotic resistance are strongly associated with the overuse and misuse of antibiotics in clinical, agricultural, and environmental settings (Spellberg and Gilbert, 2014). As resistance to conventional antibiotics continues to increase, the effectiveness of current therapeutic options is gradually declining, leaving limited treatment choices for infections caused by multidrug-resistant (MDR) bacterial pathogens. Therefore, the identification and development of alternative antimicrobial strategies are urgently needed to address the growing challenge of MDR bacteria (Salam et al., 2023). Among the emerging alternatives, bacteriophage-derived proteins, particularly endolysins, have gained considerable attention in recent years. Endolysins are peptidoglycan hydrolases that exhibit bacteriolytic activity against a wide range of bacterial pathogens, including multidrug-resistant strains. In addition, endolysins often demonstrate high specificity toward their target bacteria, which may reduce disruption of the normal microbiota compared with broad-spectrum antibiotics. Although additional studies are required to further evaluate their clinical safety, immunogenicity, and long-term therapeutic efficacy, endolysins are increasingly being explored as promising antimicrobial agents for the treatment and control of bacterial infections (Khan et al., 2023; Khan et al., 2024). Endolysins possess several potential advantages, including rapid bacterial lysis, broad lytic activity against different bacterial strains, defined pharmacokinetic properties in reported studies, and a comparatively lower likelihood of resistance development than many conventional antibiotics. Due to these characteristics, endolysins are being investigated as potential alternative therapeutic agents for the treatment of multidrug-resistant bacterial infections (Pastagia et al., 2011). One of the important features of endolysins, compared with traditional broad-spectrum antibiotics, is their specificity toward target bacteria, which may help reduce adverse effects on the normal microbiota (Schmelcher et al., 2012). The increasing prevalence of antibiotic resistance has generated considerable interest in the use of endolysins as potential alternatives or adjuncts to conventional antibiotics. Endolysins can hydrolyze bacterial peptidoglycan independently of the complete phage replication cycle; therefore, their potential applications have expanded to human medicine, veterinary medicine, and agriculture (Abdelrahman et al., 2021). However, although endolysins have shown promising antibacterial activity in several experimental studies, further investigations are still required to evaluate their long-term efficacy, immunogenicity, and possible formulation-dependent effects (Mehmood Khan et al., 2023). Most reported endolysins exhibit effective antibacterial activity against Gram-positive bacterial pathogens. In contrast, their activity against Gram-negative bacteria is often limited by the presence of the outer membrane, which restricts endolysin access to the underlying peptidoglycan layer (Briers et al., 2014). Based on their structural organization and target bacterial groups, endolysins are generally classified into Gram-positive and Gram-negative endolysins. The structure, classification, and mechanisms of action of endolysins have been comprehensively discussed in our previously published review article (Khan et al., 2023).

## 2 Endolysins as a novel therapeutic approach

Phage therapy has emerged as a potential treatment strategy for clinical infections in response to the increasing challenge of antimicrobial resistance (Mehmood Khan et al., 2023). Previous studies suggest that bacterial cell-wall components, including choline, polyrhamnose, and neutral polysaccharides, may serve as receptor-binding sites for the cell wall-binding domains (CBDs) of endolysins in different bacterial species (Schuch et al., 2002; Fischetti et al., 2006). Resistance to endolysins appears to be less frequently reported than resistance to many conventional antibiotics, although systematic long-term resistance studies remain limited. Repeated exposure of *Staphylococcus aureus, Streptococcus pneumoniae*, and *Bacillus cereus* to endolysins has generally not resulted in the emergence of stable spontaneously resistant mutants under reported experimental conditions. In contrast, substantial increases in resistance have been observed with conventional antibiotics such as streptomycin and novobiocin (Schuch et al., 2002). Similarly, *in vitro* exposure of *S. aureus* to increasing concentrations over 8 days showed that the endolysin ClyS had a lower tendency to induce resistance than mupirocin (Pastagia et al., 2011). Repeated treatment of methicillin-resistant *S. aureus* (MRSA) and methicillin-sensitive *S. aureus* (MSSA) with sub-minimum inhibitory concentrations (sub-MICs) of LysGH15 also did not lead to detectable resistance under the tested experimental conditions (Zhang et al., 2016). Although resistance to phage endolysins appears to be limited compared with conventional antibiotics, further studies are needed to clarify the mechanisms of resistance and to monitor its emergence during therapeutic development. Combining endolysins with conventional antibiotics has also shown synergistic antibacterial effects and may provide an additional strategy for treating multidrug-resistant infections. Recent studies have further highlighted the potential of protein engineering and nanotechnology to improve the stability, pharmacokinetics, delivery, antibiofilm activity, and activity of endolysins against Gram-negative bacteria (Sharma et al., 2026). Therefore, integrating resistance surveillance with mechanistic studies and emerging engineering and computational approaches may support the development of more effective endolysin-based therapies (Qian and Zheng, 2026). In addition, several studies have reported that some endolysins retain lytic activity in the presence of bacterial biofilms and polysaccharide capsules, although their antibacterial effectiveness may vary depending on the endolysin type, bacterial species, and experimental conditions used (Pastagia et al., 2013).

### 2.1 Minimal cytotoxicity and safety record

Endolysins possess significant potential for therapeutic applications due to their relatively low cytotoxicity toward human (eukaryotic) cells in reported experimental studies (Son et al., 2023). The primary target of endolysins is the bacterial peptidoglycan cell wall, which is absent in human cells. Their safety profile is mainly associated with their high specificity toward target bacteria, as endolysins specifically hydrolyze bacterial peptidoglycan. In contrast, traditional broad-spectrum antibiotics may disrupt the beneficial natural microbiota of the human body and can also cause adverse side effects (Rahman et al., 2021). Since human eukaryotic cells lack peptidoglycan, endolysins generally show low cytotoxicity toward human cell lines under reported experimental conditions. However, additional studies are still required to evaluate their immunogenicity, off-target effects, and formulation-dependent toxicity. Furthermore, even low concentrations of endolysins have demonstrated effective antibacterial activity against pathogenic bacteria (Zheng and Zhang, 2024). Due to their rapid lytic activity, specificity against multidrug-resistant bacterial pathogens, and comparatively lower likelihood of resistance development, endolysins are considered promising novel antimicrobial therapeutic agents (Belete et al., 2024).

### 2.2 Eradication of bacterial biofilms

The treatment of bacterial infections associated with biofilm formation remains a major challenge because biofilms can contribute to persistent infections and reduce susceptibility to antimicrobial agents. Therefore, the effective eradication of bacterial biofilms in hospital environments and the food industry is important for infection control and food safety (Shi and Zhu, 2009). Bacterial cells embedded within biofilms often exhibit greater pathogenicity and tolerance to antimicrobial treatment than planktonic cells (Abee et al., 2011). Several studies have investigated the potential application of endolysins for biofilm disruption and eradication. For example, the endolysin LysSTG2 demonstrated the ability to degrade *P. aeruginosa* biofilms, suggesting its potential application as a biocontrol agent in food safety and sterilization processes (Zhang et al., 2022). Similarly, *Staphylococcal* endolysins SAP-2 and Phi11 were reported to eliminate *S. aureus* biofilms formed on polystyrene surfaces (Sass and Bierbaum, 2007; Son et al., 2010). The endolysin LysH5 also exhibited lytic activity against *S. aureus* biofilms, and less detectable resistance was observed under the reported experimental conditions (Gutiérrez et al., 2014). In addition, the *staphylococcal* endolysin PlyGRCS demonstrated activity against MRSA and reduced biofilm formation (Linden et al., 2015). Another endolysin, LysCSA13, effectively reduced *Staphylococcal* biofilms on stainless steel, glass, and polystyrene surfaces, resulting in an approximately 80%–90% decrease in biofilm mass (Cha et al., 2019). LysSMP disrupted more than 80% of biofilm biomass in *Streptococcus suis* strains SS2-4 and SS2-H, among 32 clinical isolates screened for biofilm formation (Meng et al., 2011). The *Streptococcus pyogenes* endolysin PlyC was also reported to rapidly degrade biofilm matrices, including those containing antibiotic-tolerant bacterial cells (Shen et al., 2013). The eradication of bacterial biofilms in food supply chains and food-processing environments is essential for minimizing cross-contamination and ensuring food safety, as well as for the potential application of antimicrobials in food-related systems. Recent studies have investigated the use of endolysins for the control of biofilms formed by clinically and industrially relevant bacteria, including *Streptococcus, Listeria,* and *Staphylococcus* species (Son et al., 2010; Gutierrez et al., 2014; Cha et al., 2019). Gram-negative bacteria have also been investigated as targets for endolysin-mediated biofilm control. For example, the phage-derived endolysin LysSTG2 showed lytic activity against several Gram-negative bacteria, including *Salmonella, Escherichia coli, and P. aeruginosa*, following membrane permeabilization. Importantly, LysSTG2 also reduced the viability of established *Salmonella Typhimurium* biofilm cells. Sequential treatment with slightly acidic hypochlorous water and LysSTG2 resulted in more than 99% removal of *S. Typhimurium* biofilm cells, demonstrating the potential of endolysin-based approaches for controlling Gram-negative biofilms in food-related environments (Zhang et al., 2021). The endolysin LysAB1245 also exhibited antibiofilm activity against *Pseudomonas aeruginosa*, significantly reducing established biofilms in a concentration-dependent manner (Soontarach et al., 2025). The engineered hydrophobic fusion endolysin lysAB-vT2-fusion showed antibiofilm activity against mature *Acinetobacter baumannii* biofilms. Treatment with 4 mg/mL lysAB-vT2-fusion significantly reduced viable cells within mature biofilms. Its combination with the parent phage vB_AbaM_PhT2 further enhanced biofilm-associated bacterial reduction (Sitthisak et al., 2023). The engineered endolysin LysAB2-KWK also showed a prominent ability to disrupt *A. baumannii* biofilm formation, demonstrating the potential of membrane-permeable endolysins for controlling biofilm-associated multidrug-resistant bacteria (Chen et al., 2021). The endolysin Abtn-4 from phage vB_AbaP_D2 demonstrated antibiofilm activity by reducing biofilm formation by Gram-negative bacteria, including *A. baumannii.* Abtn-4 also reduced biofilm formation by some Gram-positive strains, including *S. aureus* (Yuan et al., 2021). Four endolysins—LysAm24, LysAp22, LysECD7, and LysSi3—showed antibiofilm activity against mature biofilms of *A. baumannii, Klebsiella pneumoniae,* and *P. aeruginosa*. After 2 h of treatment, all four endolysins reduced biofilm biomass in a concentration-dependent manner, with LysECD7 showing the strongest activity and reaching the reported biofilm-eradication threshold. Microscopy confirmed disruption of biofilm structure and bacterial cell damage, although some viable cells remained after treatment (Vasina et al., 2021). The endolysin LysPA26 showed activity against established *P. aeruginosa* biofilms. Treatment of 48-h-old biofilms with LysPA26 resulted in a dose-dependent reduction in biofilm biomass. At 100 μg, LysPA26 reduced viable biofilm-associated cells by approximately 1–2 log units after 2 h of treatment (Guo et al., 2017). Although these findings highlight the potential of endolysins as antibiofilm agents, their effectiveness may vary depending on bacterial species, biofilm composition, environmental conditions, and endolysin type. Therefore, additional studies are required to further evaluate their long-term efficacy, resistance potential, and applicability in clinical and industrial settings.

### 2.3 Natural bio-preservative for food chain security

Endolysins have also been investigated as natural biopreservatives for controlling foodborne pathogens in different food matrices. Their applications have been reported in dairy products, meat, vegetables, and food-processing environments, with studies demonstrating activity against pathogens such as *E. coli. S. aureus, L. monocytogenes, Salmonella Typhimurium, and Campylobacter jejuni.* These findings support the potential use of endolysins as targeted antimicrobial agents for improving food safety while reducing reliance on conventional antimicrobials (Khan et al., 2023). In addition, studies conducted *in vitro* and in food models have further demonstrated the potential of endolysins and their derivatives to reduce foodborne pathogenic bacteria and control food-associated biofilms. Their rapid and specific antibacterial activity, together with their stability under different conditions, supports their potential application as natural antimicrobials in food safety and processing environments (Aliakbarlu et al., 2025). Endolysins have also shown potential for controlling *Listeria monocytogenes* during food production and storage. Their antimicrobial activity can be combined with established preservation methods, such as nisin and high hydrostatic pressure, to enhance the control of *L. monocytogenes*. In addition, cell-binding domains derived from endolysins have been explored for the rapid and specific detection of *L. monocytogenes* using fluorescence-based methods and biosensors. Recombinant engineering approaches have further provided opportunities to improve the lytic efficiency and specificity of endolysins for different food safety applications. However, further studies are needed to evaluate their performance under different food conditions and to address standardization, regulatory requirements, and cost-effective production for large-scale food processing (Kim et al., 2025). Endolysins have been investigated for their antimicrobial potential in food-related applications. In this context, they are considered advantageous because they do not mediate horizontal gene transfer, and the likelihood of stable bacterial resistance development appears to be lower than that observed for many conventional antibiotics, although long-term systematic studies remain limited (Lee et al., 2023). Resistance to these enzymes is thought to require alterations in essential and evolutionarily conserved peptidoglycan structures; however, such mutations are considered infrequent under reported conditions (Nelson et al., 2012). Endolysins generally display targeted activity against specific bacterial species, which may help preserve beneficial microbiota in food systems. Another important characteristic is their rapid lytic activity, which can be effective at relatively low concentrations (Loessner, 2005). The application of endolysins has been explored across a range of food matrices, including dairy products, meat, vegetables, crops, and food-associated biofilms. Among these, dairy products have been most extensively studied, as controlling foodborne pathogens in dairy processing environments is critical for food safety (Oliver et al., 2005). Several endolysins have demonstrated activity against *S. aureus* in milk, while others have shown antibacterial effects against *L*. *monocytogenes* and *Clostridium sporogenes* in dairy products such as milk and Queso Fresco cheese (Mayer et al., 2010; Schmelcher et al., 2012; Zhang et al., 2012; Ibarra-Sánchez et al., 2018). In meat products, which are highly susceptible to contamination due to their neutral pH, high water activity, and rich nutrient composition (Bantawa et al., 2018). Limited studies have evaluated the efficacy of endolysins against MRSA, including applications in contaminated ham models (Chang et al., 2017). In addition, endolysin-based approaches have been explored for controlling foodborne pathogens in vegetables, where activity against *L. monocytogenes* has also been reported (Schmelcher et al., 2012). Overall, endolysin-based strategies are being investigated as potential tools for reducing antibiotic-resistant foodborne pathogens in food production systems.

### 2.4 Veterinary medicine: alternative to antibiotics

The misuse and overuse of antibiotics in livestock production and the food industry have raised significant concerns, driving the search for alternative antimicrobial strategies. In this context, endolysins have been investigated as potential agents against multidrug-resistant bacterial infections in animals (Love et al., 2018). *Clostridium perfringens,* a major causative agent of necrotic enteritis and subclinical disease in poultry, is associated with considerable economic losses in the poultry industry (Timbermont et al., 2011). This pathogen is also recognized as one of the leading contributors to foodborne illness in humans. Therefore, endolysins have been explored as potential antibiotic alternatives in poultry production. For example, the thermostable chimeric endolysin PlyGVE2CpCWB has shown potential as a feed additive for bacterial control in chicken production systems (Swift et al., 2015). Bovine mastitis, a major economic burden in dairy farming, is primarily caused by bacterial infections, with *streptococci* and *staphylococci* accounting for approximately 75% of cases (Wilson et al., 1997). Endolysin-based treatments have been reported to reduce *S. aureus*-associated bovine mastitis (Schmelcher et al., 2012). Anthrax, caused by *B. anthracis*, is a severe zoonotic disease affecting both animals and humans (Inglesby et al., 2002). The endolysin PlyG has demonstrated activity against *Bacillus anthracis in vitro* and *in vivo*, including its spores, suggesting its potential as a biocontrol agent for this pathogen (Schuch et al., 2002). *Streptococcus equi* is an important equine pathogen that causes strangles, a disease characterized by upper respiratory tract infection followed by abscess formation in retropharyngeal lymph nodes (Boyle et al., 2018). The endolysin PlyC has shown efficacy against *S. equi* and has also been evaluated as a disinfectant for controlling pathogen transmission, with reported effectiveness comparable to the commonly used disinfectant Virkon-S (Hoopes et al., 2009). Endolysins have also been investigated for applications in veterinary medicine. For example, the bacteriophage-derived chimeric protein P128 showed antistaphylococcal activity against clinical *S. aureus* isolates, including methicillin-resistant and methicillin-sensitive strains. *In vitro* testing showed that P128 had MIC and MBC values ranging from 1 to 64 μg/mL across 32 *S. aureus* strains, and time-kill assays showed a 99.99% reduction in viable cells within 1 h (Vipra et al., 2012). P128 was subsequently evaluated in dogs with pyoderma. Among 87 samples collected from affected dogs, 46 *Staphylococcus* strains were isolated, and all were susceptible to P128 despite resistance to different antibiotics. In a clinical study involving 17 dogs with pyoderma, topical application of P128 hydrogel twice daily for 8 days resulted in complete healing of the treated lesions (Junjappa et al., 2013). *Clostridioides difficile* is another bacterial pathogen of veterinary relevance. Debast et al. (2009) identified *C. difficile* PCR ribotype 078 in diarrhoeal piglets and found that the porcine isolates were closely related to isolates obtained from affected humans. This finding supports the relevance of *C. difficile* as a target in animal health. In this context, Mayer et al. (2008) characterized the bacteriophage ΦCD27 endolysin CD27L and demonstrated activity against all 30 *C. difficile* strains tested, including epidemic ribotype 027 strains. Importantly, CD27L showed little or no activity against several commensal bacteria from the gastrointestinal tract, indicating a relatively narrow spectrum of activity toward *C. difficile* (Mayer et al., 2008). Thus, the veterinary relevance of *C. difficile* and the species-specific activity reported for CD27L provide a basis for considering endolysin-based approaches for targeted control of this pathogen. Endolysin-based approaches have also been investigated against *Salmonella*, an important pathogen in veterinary and food-production settings. Ding et al. (2020) characterized LysSE24 from bacteriophage LPSE1 and reported specific bactericidal activity against *Salmonella*, including multidrug-resistant strains. LysSE24 showed bactericidal activity against 23 tested multidrug-resistant *Salmonella* strains, and its activity against *S. Enteritidis* was enhanced in the presence of an outer-membrane permeabilizer. The enzyme also retained activity under a range of pH and temperature conditions, supporting further investigation of LysSE24 as an antimicrobial approach against *Salmonella* (Ding et al., 2020). Overall, these studies demonstrate that phage-derived lytic proteins have been experimentally investigated against bacterial pathogens relevant to veterinary medicine, including *Staphylococcus*, *C. difficile*, and *Salmonella*. Their reported activity in animal-associated isolates and, in the case of P128, in dogs with pyoderma, supports continued investigation of endolysin-based approaches as alternatives to conventional antibacterial treatments. Figure 1 summarizes the applications and advantages of phage-derived endolysins.

**FIGURE 1:**
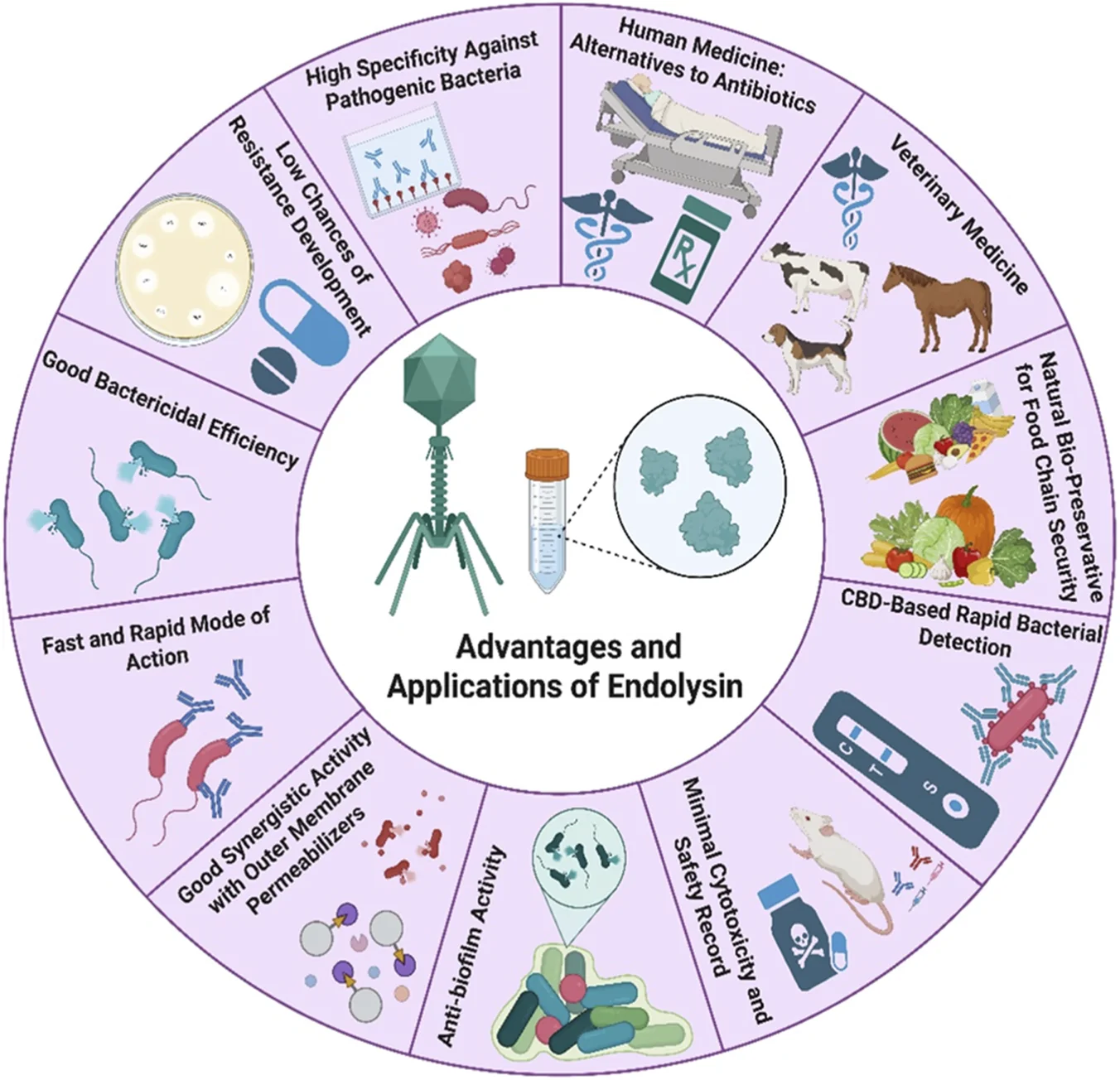
Overview of the applications and advantages of phage-derived endolysins. Image was created by BioRender.com.

## 3 The role of endolysin in diagnostic and detection

Rapid and accurate detection of pathogenic bacteria is essential in clinical settings, particularly for MDR pathogens such as MRSA, where delayed diagnosis may lead to severe infections, nosocomial transmission, and increased mortality in immunocompromised patients. Traditional culture-based diagnostic methods are time-consuming, resource-intensive, and require trained personnel and multiple reagents (Quintela et al., 2022). Molecular diagnostic techniques such as PCR, LAMP, NASBA, and microarrays provide high sensitivity and specificity; however, they require expensive equipment, nucleic acid extraction, and trained operators. In addition, these methods cannot reliably distinguish between viable and non-viable cells or between target and non-target microorganisms. Immunological methods are comparatively less expensive but often suffer from lower sensitivity and require pre-enrichment steps (Canciu et al., 2021; Wang et al., 2023). To address these limitations, phage-derived proteins have been increasingly explored as alternative diagnostic tools due to their strong specificity and ability to recognize bacterial targets.

### 3.1 Endolysin-derived cell wall binding domains (CBDs)

Endolysin-derived cell wall binding domains (CBDs) have emerged as important recognition elements for bacterial detection. CBDs specifically bind to ligands present in bacterial peptidoglycan, enabling selective interaction with target cells. This binding property has been widely exploited for pathogen identification, particularly in food and clinical samples. For example, endolysins such as LysK and Φ11 have been shown to bind specifically to *Staphylococcus* cells, demonstrating their potential in pathogen detection systems (Chibli et al., 2014). In clinical applications, CBD-based systems have been used for the rapid detection of *S. aureus* in blood samples using flow cytometry, highlighting their diagnostic utility in bloodstream infections (Costa et al., 2020). Currently, scientists are focusing on the use of phage-derived endolysin-based biosensors for the diagnosis and detection of pathogenic bacteria to overcome the barriers and limitations of conventional detection techniques. These endolysin-based probes have demonstrated reasonable specificity and sensitivity for pathogen identification. They have been reported to enable rapid detection of bacterial cells, with potential for application in systems that reduce or eliminate the need for pre-enrichment steps (Aliakbar Ahovan et al., 2020; Quintela et al., 2022). CBDs, therefore, provide a selective recognition platform for bacterial detection based on specific cell wall interactions.

### 3.2 Phage receptor-binding proteins (RBPs), tail fibers, and tail spike proteins

In addition to CBDs, bacteriophage receptor-binding proteins (RBPs), including tail fibers and tail spike proteins, play a central role in bacterial recognition. RBPs are structural components located at the phage tail and are responsible for host recognition and initial attachment prior to infection (Nobrega et al., 2018). Recent ICTV taxonomy updates have established a genome-based classification framework for bacterial viruses and abolished the former morphology-based families Podoviridae*,* Siphoviridae, and Myoviridae, as well as the order *Caudovirales.* The updated taxonomy also introduced a binomial nomenclature system for bacterial virus species (Turner et al., 2023). Long tail fibers (LTFs), often forming trimeric filamentous structures, mediate reversible binding to bacterial surface receptors, including outer membrane proteins, pili, flagella, lipopolysaccharides (LPS), and teichoic acids (Klumpp et al., 2023; Walter et al., 2008). Following this reversible interaction, structural rearrangements in the phage baseplate facilitate stronger attachment through short tail fibers (STFs), leading to irreversible binding (Dunne et al., 2018). Tail spike proteins (TSPs) are also important receptor-binding structures that commonly recognize bacterial surface polysaccharides. In some cases, TSP-associated enzymatic activities (e.g., lyases, glycosidases, or esterases) modify the LPS O-antigen, strengthening irreversible phage attachment. However, host specificity and binding domains vary among different TSPs (Choi et al., 2025). These RBPs and tail-associated proteins function as highly specific molecular recognition elements and have been explored for diagnostic applications due to their ability to bind bacterial surface receptors with high selectivity.

### 3.3 Reporter-fused phage proteins as biosensors

Phage-derived binding proteins, including CBDs and RBPs, can be genetically engineered and fused with reporter molecules to enhance bacterial detection. These reporter systems enable optical or enzymatic signal generation upon binding to target bacteria. For instance, fusion of the endolysin CBD with green fluorescent protein (GFP) enabled the detection of *Clostridium perfringens* in milk samples (Cho et al., 2021). Similarly, spectrofluorometric detection systems using phage tail proteins have been developed for *S. aureus* and *Enterococcus faecalis* (Santos et al., 2020). Bioluminescent reporter systems have also been applied, such as the fusion of phage gp37 with NanoLuc luciferase (NLuc-LTF), which successfully bound to multiple *Escherichia coli* strains and generated detectable fluorescence signals (Farquharson et al., 2021). In addition, ELISA-based platforms have been developed using CBDs or tail fibers for detection of pathogens such as *S. aureus* and *Yersinia enterocolitica* (Filik et al., 2022). These engineered reporter systems have also been applied to detect foodborne pathogens in complex matrices such as lettuce, meat, and poultry, demonstrating their potential for food safety applications (Chen et al., 2023).

### 3.4 CBD-based detection in food safety applications

CBD-based systems have been widely investigated for detecting foodborne pathogens in food supply chains. Compared with antibodies, CBDs offer high stability, strong binding specificity, and ease of recombinant production. In food diagnostics, CBDs function through specific binding to bacterial peptidoglycan, enabling selective detection of pathogens in complex food matrices. CBD-coated magnetic beads have been used to recover more than 90% of *L. monocytogenes* from contaminated samples (Kretzer et al., 2007). Similarly, CBD-functionalized nanoparticles have been used to detect *Bacillus cereus* in biological samples with minimal cross-reactivity (Park et al., 2018).Fluorescently labeled CBDs have also been used to detect *Listeria* species in dairy products such as milk and cheese (Schmelcher et al., 2010). In addition, CBDs derived from the CTP1L endolysin have been used for detection of *Clostridium tyrobutyricum* in cheese (Gómez-Torres et al., 2019). The CBD-coated beads detected the *S. aureus* in the milk sample. Integration of CBD-coated beads with qPCR enabled sensitive detection, achieving a detection limit of 2 CFU/mL in PBS and less than 10 CFU/mL in milk or beef extract (Kwon et al., 2018). Overall, CBD-based biosensors provide rapid, specific, and low cross-reactive detection systems, representing a promising alternative to antibody-based diagnostics. However, further optimization and validation are still required for large-scale applications in food safety monitoring (Agún et al., 2025). Figure 2 illustrates the rapid detection and identification of bacterial pathogens using phage-derived detection systems.

**FIGURE 2:**
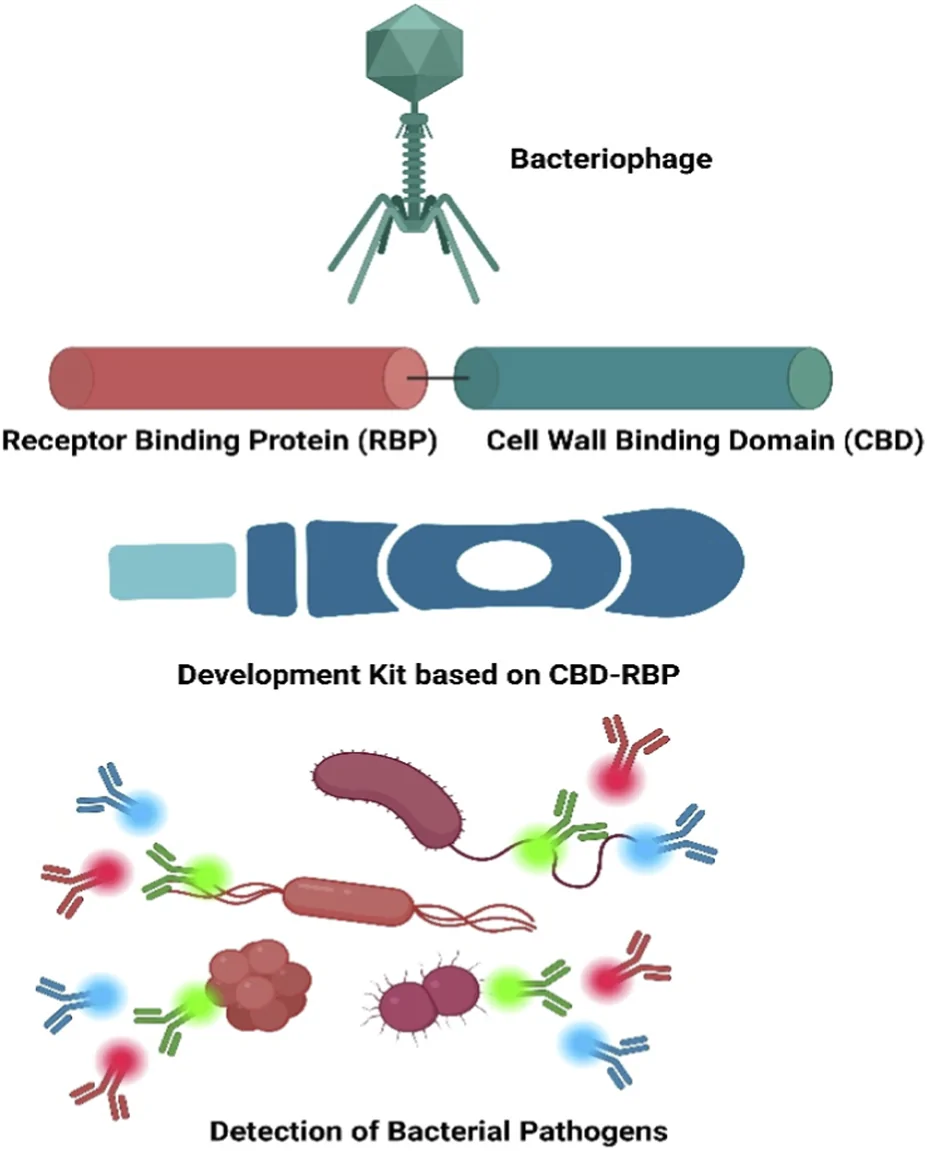
Schematic representation of phage-derived detection systems for bacterial detection. Bacteriophages possess bacterial-specific binding proteins that recognize surface receptors such as teichoic acids and lipopolysaccharides (LPS). Phage-derived proteins, including endolysin cell wall binding domains (CBDs) and receptor-binding proteins (RBPs), can be utilized to develop specific bacterial detection platforms and biosensor systems. Image was created by BioRender.com.

## 4 Primary obstacles of endolysins

### 4.1 Challenges in the expression of endolysin

The discovery of novel endolysins requires several experimental steps, including bacteriophage isolation, purification, genome extraction, sequencing, sequence analysis, cloning of endolysin-encoding genes, transformation, recombinant expression, purification, and characterization. However, recombinant expression of endolysins can be affected by their low solubility, which may limit the recovery of soluble enzyme (Zheng and Zhang, 2024). Balaban et al. (2022) systematically evaluated factors affecting the expression and extraction of recombinant endolysins in *E. coli*. They found that induction at a lower temperature (20 °C) improved the recovery of soluble enzyme, whereas changing the concentration of isopropyl β-D-1-thiogalactopyranoside (IPTG) from 0.1 to 1 mM did not appreciably change endolysin yields. The study also showed that the position of the hexahistidine (His) tag and additional amino acid residues introduced during cloning could negatively affect recombinant endolysin recovery. Fusion with solubility-enhancing proteins, including thioredoxin (Trx), disulfide bond isomerase C (DsbC), and small ubiquitin-like modifier (SUMO), did not increase the yield of recombinant endolysins under the conditions tested. Similarly, co-expression of molecular chaperones did not improve endolysin yield (Balaban et al., 2022). The study further showed that the composition of the lysis buffer can affect endolysin recovery. Addition of 25% glycerol, 0.75 M trehalose, or 1 M glycine betaine improved endolysin recovery, while the anionic detergent N-lauroylsarcosine produced the most noticeable improvement in soluble protein yield without affecting enzymatic activity under the tested conditions (Balaban et al., 2022). These findings indicate that expression temperature, construct design, and extraction conditions can influence the recovery of soluble recombinant endolysins and should therefore be considered when optimizing their production.

### 4.2 Hurdles in the cloning of endolysin

Cloning endolysin genes into heterologous expression vectors requires careful design of the recombinant construct. The choice of restriction sites and cloning strategy can influence the sequence of the resulting recombinant protein by introducing additional residues or altering the position of affinity tags. These changes may affect the properties of the cloned endolysin. For example, Tham et al. (2020) compared two cloning strategies for a staphylococcal phage endolysin using the pET-28a vector. Cloning through the BamHI/XhoI sites resulted in a recombinant protein containing additional N-terminal sequences, whereas cloning through the NcoI/XhoI sites removed these additional residues and produced a different construct. The two constructs showed different outcomes after recombinant production, with the NcoI/XhoI construct yielding a soluble and functional endolysin, whereas the BamHI/XhoI construct produced protein that accumulated in inclusion bodies and remained nonfunctional after solubilization (Tham et al., 2020). These findings indicate that cloning design, including the selection of cloning sites and the resulting terminal sequence or tag configuration, should be carefully considered when constructing recombinant endolysin genes. Verification of the cloned insert by DNA sequencing is also important to confirm the intended sequence before subsequent characterization.

### 4.3 Challenges associated with the costly production of endolysin

Bacteriophage-derived endolysins exhibit promising lytic activity; however, their expression, purification, and characterization require complex experimental procedures and protocols. In addition, subsequent *in vitro* and *in vivo* evaluations are time-consuming and require substantial laboratory resources, which may increase overall production costs and limit their broader application in the food industry and aquaculture sectors (Xu, 2021). The high production cost remains a major challenge for the development of endolysin-based diagnostic kits, hospital-associated detection systems, and strategies for controlling food-borne pathogens within food supply chains. Endolysins are commonly produced using bacterial or yeast expression systems, and the purification process, including the removal of contaminating host proteins, can be technically demanding and costly. Furthermore, the translation of endolysins from laboratory research to commercial applications requires extensive validation in animal models and experimental trials, which further increases development costs.

### 4.4 Reduced activity under challenging conditions

The activity of endolysins can be affected by the conditions in which they are applied. For example, Guo et al. (2017) evaluated the effects of temperature, pH, and NaCl concentration on the activity of the Gram-negative endolysin LysPA26. LysPA26 showed high antibacterial activity at 37 °C–50 °C, whereas its activity decreased at temperatures below 25 °C or above 60 °C. NaCl concentrations below 150 mM had little effect on its activity, whereas 300 mM NaCl reduced its antibacterial activity. LysPA26 was active at pH 6.0–8.0, with the highest activity at pH 8.0 (Guo et al., 2017). These results show that environmental conditions can affect the activity of an individual endolysin, although the effects may differ among enzymes. The activity of some endolysins can also be affected when tested in complex biological matrices. Yang et al. (2015) reported that a peptide-modified lysin showed strong antibacterial activity against logarithmic-phase *Acinetobacter baumannii* and *P. aeruginosa* in buffer, whereas no antibacterial activity was observed when the lysin was tested in culture medium, milk, or human serum. The authors suggested that components of these matrices might affect the membrane-penetrating activity of the modified lysin, although this mechanism was not directly established in the study (Yang et al., 2015). A similar observation was reported for the *A. baumannii* phage endolysin LysAB54. Khan et al. (2021) found that LysAB54 showed strong antibacterial activity in buffer but substantially reduced activity when tested in LB medium and human serum. The authors suggested that components of complex media, including ions, might interfere with the interaction between LysAB54 and the bacterial outer membrane; however, this proposed mechanism was not experimentally confirmed (Khan et al., 2021). These findings indicate that the activity of an endolysin should be evaluated under the environmental or biological conditions relevant to its intended application. Importantly, the effect of such conditions is not necessarily the same for all endolysins and may depend on the enzyme, target bacterium, and surrounding matrix. Therefore, results obtained under controlled *in vitro* conditions should be interpreted carefully when considering potential therapeutic or biocontrol applications.

### 4.5 Sequence-based prediction and experimental validation of endolysin activity

Bioinformatic analysis can be used to identify putative endolysins and predict their conserved functional domains. Kim and Seo (2023) developed a bioinformatic workflow to identify endolysin candidates against *S. aureus*. They identified 114 putative endolysins and grouped them according to their conserved domain combinations. Two candidates were selected for further study based on predicted protein solubility, but only one, LyJH1892, was successfully overexpressed. LyJH1892 was subsequently shown to have lytic activity against methicillin-susceptible *S. aureus* and methicillin-resistant *S. aureus* (Kim and Seo, 2023). Sequence and structural analyses can also provide information about endolysin domain organization. Khan et al. (2024) screened *S. pneumoniae* phage genomes and curated 89 lytic proteins from 81 phage genomes. These proteins were classified into eight groups according to their enzymatically active domains and cell wall-binding domains. The study also identified an additional cell wall-binding repeat in a Group II endolysin and predicted cell wall-binding domains in Group III endolysins using comparative computational analyses (Khan et al., 2024). Experimental studies have further shown that structural regions outside the catalytic domain can affect endolysin properties. Munetomo et al. (2024) examined linker-deletion mutants of the *Staphylococcus* phage S6 endolysin S6_ORF93. Except for the Δ20 mutant, the wild-type protein and the linker-deletion mutants were expressed as soluble proteins. The Δ15 mutant showed the highest protein yield and bacteriolytic activity among the tested proteins, while its antimicrobial spectrum was similar to that of the other tested proteins (Munetomo et al., 2024). Structural and mutational analyses of the broad-spectrum endolysin Ply2741 also identified specific residues involved in its activity. Wang et al. (2025) reported that the bactericidal activity of Ply2741 depended on a Cys-His-Asn catalytic triad. Their site-directed mutagenesis experiments further showed that Gln29 contributed to lytic activity, while R189 and W250 in the cell wall-binding domain were involved in peptidoglycan binding (Wang et al., 2025). These studies show that sequence-based analysis is useful for identifying candidate endolysins and their functional domains, while experimental characterization is required to determine the activity of individual candidates and to examine the contribution of specific structural regions to their function (Aitken et al., 2025).

### 4.6 The outer membrane of gram-negative bacteria as a barrier to endolysin activity

Endolysins exhibit different antibacterial activities against Gram-positive and Gram-negative bacteria due to differences in their cell envelope structures. In general, endolysins show stronger bactericidal activity against Gram-positive bacteria, whereas their activity against Gram-negative bacteria is often limited. This reduced activity is mainly associated with the outer membrane of Gram-negative bacteria, which acts as a protective permeability barrier and restricts endolysin access to the peptidoglycan layer. The outer membrane is composed of lipopolysaccharides (LPS), phospholipids, and outer membrane proteins that limit the penetration of externally applied endolysins and reduce their interaction with peptidoglycan. Consequently, many reported endolysins show only moderate lytic activity against Gram-negative bacteria, commonly resulting in approximately 1–2 log reductions in bacterial counts under reported experimental conditions. (Lai et al., 2020). Some naturally occurring Gram-negative endolysins possess structural characteristics that may facilitate partial interaction with or penetration across the outer membrane. In contrast, engineered endolysins are commonly modified by fusion with outer membrane-permeabilizing peptides or other functional domains to improve their access to peptidoglycan and enhance antibacterial activity against Gram-negative pathogens. However, the effectiveness of these strategies may vary depending on the bacterial species, environmental conditions, and physiological media. In addition, several Gram-negative endolysins have shown reduced activity in complex biological conditions, including human serum (Sisson et al., 2024) Although phage-derived endolysins demonstrate promising antibacterial potential, several biological and technical limitations remain to be addressed before their broader clinical and diagnostic applications can be achieved. A comparative overview of representative endolysins, their applications, and limitations is provided in Table 1. Figure 3 illustrates the major challenges and limitations associated with endolysin applications.

**TABLE 1:** Summary of selected phage-derived antibacterial proteins.

Endolysin name	Target bacterial pathogen	Application	Experimental model	Main advantages	Current challenges and limitations	References
• LysAb54	• MDR *A. baumannii* • *P. aeruginosa* • *K. pneumoniae* • *E. coli*	• Potential alternative antimicrobial against MDR Gram-negative bacteria • Potential application against topical infections, as suggested by the authors	• Recombinant LysAB54 was expressed and purified from *E. coli* • *In vitro* antibacterial activity was evaluated in Tris buffer using both logarithmic- and stationary-phase cells • At 100 μg/mL, LysAB54 produced an approximately 4-log_10_ reduction in logarithmic-phase *A. baumannii* within 10 min • Activity was also evaluated in LB broth and serum	• Bactericidal activity against several MDR Gram-negative bacteria • Activity without an outer-membrane permeabilizer • Active against both logarithmic- and stationary-phase cells • Rapid activity against *A. baumannii*	LysAB54 completely lost antibacterial activity in LB broth and serum • Activity was demonstrated only under *in vitro* conditions • Biofilm and animal infection models were not evaluated • Further validation in biofilm and animal models is required	(Khan et al., 2021)
• LysP53	• MDR *A. baumannii* *• P. aeruginosa* *• K. pneumoniae* *• E. coli*	• Potential broad-spectrum antimicrobial against MDR Gram-negative bacteria • Potential treatment of topical infections, including burn wounds	• *In vitro* antibacterial assays against antibiotic-resistant Gram-negative bacteria • Mouse *A. baumannii*-associated burn infection model • Cytotoxicity was also evaluated experimentally	• Broad-spectrum activity against multiple antibiotic-resistant Gram-negative bacteria •Approximately 5-log_10_ reduction of viable *A. baumannii* after 100 μg/mL LysP53 for 1 h *in vitro* • Contains a functional N-terminal 33-aa antimicrobial peptide with activity comparable to LysP53 • A single 14 µg/mouse dose produced approximately 3-log_10_ bacterial reduction in the mouse burn model	• Activity and therapeutic efficacy were primarily demonstrated in in vitro assays and a mouse burn-infection model • Further studies are required to establish efficacy and safety in clinical settings • Pharmacokinetic properties and clinical translation remain to be evaluated	(Li et al., 2021)
• ClyS	• Methicillin-resistant *Staphylococcus aureus* (MRSA) • Methicillin-sensitive *Staphylococcus aureus* (MSSA)	• Potential topical agent for *S. aureus* skin decolonization • Potential alternative to conventional topical antibacterials such as mupirocin	• *In vitro* antibacterial and resistance-development assays • Mouse skin colonization/infection model • Topical ClyS formulated as an ointment and applied to colonized/infected skin	• *Staphylococcus-*specific activity • Produced approximately 3-log_10_ reduction of *S. aureus* in the mouse model, compared with approximately 2-log_10_ reduction with mupirocin • Lower potential for resistance development than mupirocin in the *in vitro* assays • Repeated exposure did not result in inhibition of ClyS activity by antibodies generated against the enzyme	• Evidence was obtained from *in vitro* studies and a mouse skin model • Human clinical efficacy and safety have not been established • Further studies are required to evaluate clinical application and pharmacokinetic properties	(Pastagia et al., 2011)
• LysSTG2	• *P. aeruginosa* • *Pseudomonas putida*	• Potential biocontrol agent for controlling *Pseudomonas* in food products • Potential application for controlling biofilms on food-contact surfaces	• *In vitro* assays using bottled water, pasteurized milk, chicken breast, and salmon • Biofilm models using polystyrene and stainless-steel surfaces • LysSTG2 was tested alone and in combination with EDTA	• Activity against both planktonic and biofilm cells • In bottled water, LysSTG2 with EDTA reduced *P. aeruginosa* by 2.2 log_10_ and reduced *P. putida* to below the detection limit • LysSTG2 showed more than 99.9% reduction of biofilm cells on polystyrene and stainless-steel surfaces after 2 h • Demonstrated activity in several food matrices	• No significant reduction of *Pseudomonas* was observed in pasteurized milk • Further optimization is required for application in different food matrices • Evidence is currently based on laboratory-scale models • Further evaluation is required for practical food-processing applications	(Zhang et al., 2022)
• LysH5	• *S. aureus* • *S. epidermis*	• Potential antibiofilm agent • Potential application for controlling persistent *staphylococcal* contamination in food-processing environments • Potential adjuvant for treatment of chronic infections	• *In vitro* biofilm assays using 6 *S. aureus* and 3 *S. epidermidis* strains • Antibiotic-induced persister-cell assays using rifampicin and ciprofloxacin • Microscopic analysis of treated biofilms	• Reduced viable biofilm-associated cells by 1–3 log_10_ at 0.15 µM • Active against biofilms formed by both *S. aureus* and *S. epidermidis* • Eliminated persister cells selected by rifampicin and ciprofloxacin • Surviving cells after treatment did not show increased resistance to LysH5 • Sub-inhibitory concentrations did not induce biofilm formation	• The proposed food-industry application required a two-step treatment lasting approximately 18 h • The authors noted that the treatment time should be shortened for practical industrial use • Activity should be further confirmed in dynamic biofilm models • Evidence is currently based on laboratory *in vitro* models	(Gutierrez et al., 2014)
• LysK	• *S. aureus* • *S. epidermis* • MRSA	• Potential recognition element for staphylococcal biosensors • Potential application in rapid detection of *S. aureus* and other staphylococci	• *In vitro* clearing assays against *S. aureus*, MRSA isolates, and *S. epidermidis* • LysK immobilized on PEG-functionalized silicon wafers • Bacterial binding evaluated by fluorescence microscopy • Detection limit determined using *S. aureus* suspensions	• Specific binding to staphylococci with little or no binding to tested *E. coli* and *Enterococcus* controls • Detection of *S. aureus* at approximately 5,000 CFU/mL using fluorescence microscopy • Immobilized LysK provided approximately 3 bound cells/100 μm^2^ under the reported conditions • Small protein size offers advantages for surface-based biosensor platforms	• Immobilized LysK retained lytic activity, causing bacterial deformation and fragmentation that could complicate cell counting • Some binding to *Micrococcus* was observed, indicating that specificity was at the genus rather than strict species level • Further optimization is required to prevent lysis and improve signal stability for practical biosensor applications • Alternative signal-transduction methods and improved detection sensitivity require further investigation	(Chibli et al., 2014)
• GP37	• *E. coli*	• Potential recognition element for phage-based biosensors and diagnostic platforms • Characterization of phage–bacterium adsorption interactions	• *In vitro* luminescent flow-through 96-well plate assay • NanoLuc-GP37 fusion protein evaluated against the 72-strain *E. coli* ECOR collection • Binding assessed by measuring bioluminescence after filtration and washing	• GP37 adsorbed to 61 of 72 *E. coli* strains (85%) • Allowed evaluation of the adsorptive host range independently of complete phage replication • Distinguished bacterial strains that supported adsorption from those that did not support T4 plaque formation • Provides a rapid, high-throughput approach for studying phage receptor-binding interactions	• Protein aggregation occurred during storage within 24 h and required centrifugation before experiments • The assay measures adsorption rather than complete phage infection or bacterial killing • Further optimization is required for application to other phage receptor-binding proteins and diagnostic platforms	(Farquharson et al., 2021)
• CTP1L	• *C. tyrobutyricum*	• Potential fluorescence-based tool for monitoring *C. tyrobutyricum* during cheese ripening • Potential application for detection of bacterial contamination in cheese	• *In vitro* fluorescence-based binding assays • Fluorescent CTP1L cell wall-binding domain evaluated during cheese ripening • Microscopic observation of labeled bacterial cells in cheese samples	• Specific binding to *C. tyrobutyricum* in the tested cheese model • Enabled fluorescence-based detection and visualization of *C. tyrobutyricum* cells • Provided a tool for monitoring the presence and distribution of the bacterium during cheese ripening • Demonstrated application of an endolysin cell wall-binding domain directly in a food matrix	• Evaluation was performed under controlled laboratory cheese-ripening conditions • The approach was demonstrated for detection and monitoring rather than bacterial inactivation • Further validation is required under industrial cheese-production conditions • Detection performance in more complex or variable cheese matrices requires further investigation	(Gómez-Torres et al., 2019)
• LysSA11	• *S. aureus*	• Potential biocontrol agent for controlling *S. aureus* in food • Potential application for decontamination of food-contact utensils and surfaces • Potential alternative approach for food safety	• *In vitro* antibacterial assays • *S. aureus*-contaminated food samples • Contaminated kitchen utensils and food-contact surfaces • Evaluation of bacterial reduction following LysSA11 treatment	• Rapid lytic activity against *S. aureus* • Demonstrated antibacterial activity in both food and surface-contamination models • Showed potential for reducing *S. aureus* contamination in food-related environments • Provides an alternative approach to conventional chemical disinfectants and preservatives	• Evidence is based on laboratory-scale food and surface models • Further evaluation is required under industrial food-processing conditions • Effects of different food matrices and environmental conditions require further investigation • Commercial application and large-scale production require additional validation	(Chang et al., 2017)
• ZAM-CS	• MRSA. • MSSA	• Potential alternative antimicrobial against *S. aureus* • Potential antibiofilm application	• *In vitro* lytic activity assays against MRSA and MSSA. • *S. aureus* biofilm assays • Stability assays under different temperature, pH, and NaCl conditions • Scanning electron microscopy to examine morphological changes after treatment	• Rapid lytic activity against both MRSA and MSSA. • Demonstrated activity against *S. aureus* biofilms • Retained activity under a range of temperature conditions • Stable over a broad pH range • Maintained activity under different NaCl concentrations	• Efficacy was evaluated using *in vitro* models only • *In vivo* antibacterial and antibiofilm efficacy remains to be established • Further studies are required to evaluate safety and therapeutic performance under physiological conditions	(Ahmadbeigi et al., 2025)

**FIGURE 3:**
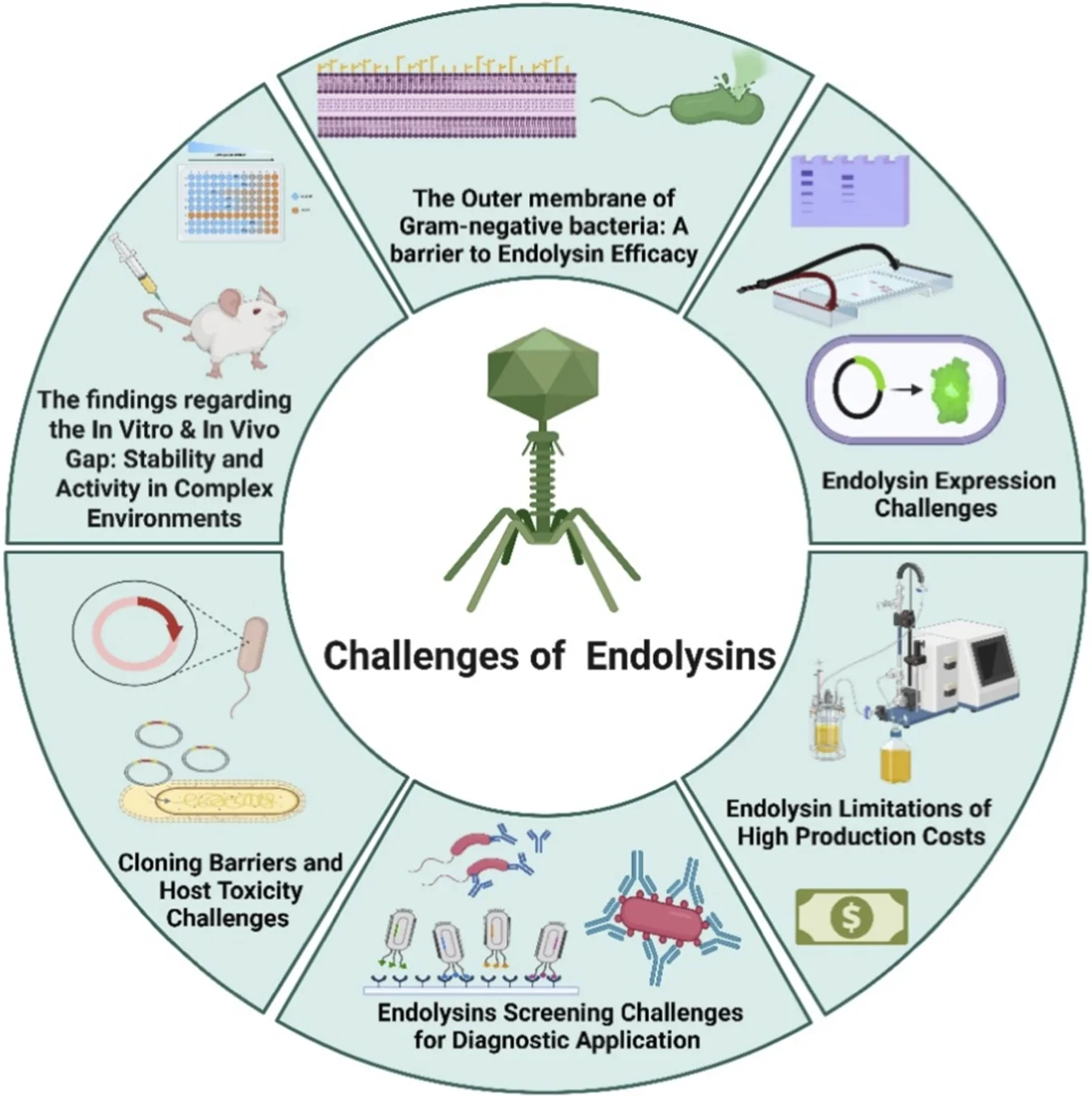
Illustrates the major challenges and limitations associated with endolysin applications. Image was created by BioRender.com.

## 5 Overcoming challenges: moving endolysins from the laboratory to practical applications

Recent advances in protein engineering have provided several approaches to improve the antimicrobial activity of endolysins against multidrug-resistant organisms. These approaches include fusion with membrane-permeabilizing peptides, domain swapping, and modification of catalytic sites. In addition, *in silico* design, library-based screening, artificial intelligence, and structural biology are increasingly being used to identify and optimize endolysin variants with improved antimicrobial properties (Aitken et al., 2025).

### 5.1 Advanced expression and production system

Bacterial expression systems, particularly *E. coli*, are widely used for the expression and production of phage-derived endolysins (Gutiérrez et al., 2018). However, purification of endolysins using the *E. coli* expression system presents several limitations, including the requirement for endotoxin removal and the difficulty of obtaining active and highly concentrated purified proteins. In addition, improper protein folding during expression may result in reduced activity or protein inactivation (Mamat et al., 2015; Balaban et al., 2022). To address these limitations, alternative expression systems and optimized culture conditions have been investigated. The endolysin LysSA11 was successfully expressed using the yeast surface display system of *Saccharomyces cerevisiae*. The expressed LysSA11 did not require an additional purification process and exhibited lytic activity against *S. aureus*. Furthermore, LysSA11 demonstrated improved stability and activity compared with proteins produced using the *E. coli* expression system (Chun et al., 2020). This yeast-based expression system offers several advantages, including reduced endotoxin contamination, simpler culture conditions, improved protein stability, and enhanced protein folding efficiency (Teymennet-Ramírez et al., 2022). Lactic acid bacteria have also been explored as alternative expression hosts for endolysin production. These bacteria are commonly used as probiotics in dairy products and may contribute to the prevention of contamination by pathogenic bacteria and bacterial toxins (Haddad Kashani et al., 2018). *Lactococcus lactis* has been utilized as a safe expression system for staphylococcal endolysins. To overcome the solubility limitations associated with the *E. coli* system, *Lactococcus lactis* was used for the expression of the staphylococcal endolysin LysK. The expressed and purified LysK demonstrated strong bactericidal activity, resulting in approximately 99% lysis of MRSA strains. (O'Flaherty et al., 2005). In addition, *L. lactis* has been used for the successful expression of other active staphylococcal endolysins, including LysH5 and Endo88 (Rodríguez-Rubio et al., 2012; Chandran et al., 2022). The application of endolysins as food preservatives for food safety and as therapeutic agents in healthcare settings requires further investigation of important factors, including endolysin stability, large-scale production, safety evaluation, and the development of reliable and safe expression systems (Liu et al., 2023).

### 5.2 Formulation and engineering strategies for enhanced stability

Bacteriophages and bacteriophage-derived endolysins offer several advantages as alternative antibacterial agents; however, they also face multiple challenges within host systems. These challenges include rapid clearance by the reticuloendothelial system, inactivation by host immune responses and antibodies, reduced activity due to neutralizing factors, and non-specific distribution within the body (Loh et al., 2021). To improve targeted delivery and stability, encapsulation-based strategies have been investigated for endolysin administration. Encapsulation within nanoparticles may protect endolysins from environmental and physiological conditions, including variations in pH and temperature, as well as exposure to different host-derived molecules such as enzymes, cytokines, and signaling factors. Liposome-mediated delivery has also emerged as a promising approach for enhancing endolysin activity, particularly against Gram-negative bacteria (Gondil and Chhibber, 2021). In one study, incorporation of the endolysin LysRODI into pH-sensitive liposomes facilitated the degradation of *S. aureus* biofilms at pH 5 (Portilla et al., 2020). Similarly, encapsulation of BSP16Lys within liposomes enabled targeted delivery and release near the bacterial cell wall. The liposome-encapsulated BSP16Lys demonstrated improved antibacterial activity against Gram-negative bacteria compared with free endolysin, likely due to enhanced interaction with the bacterial outer membrane barrier (Bai et al., 2019). Another phage-derived endolysin, Lysqdvp001, was also successfully encapsulated in liposomes, where it exhibited improved stability and bactericidal activity. In addition, the liposome formulation inhibited the growth of *Vibrio parahaemolyticus* in live clams, thereby reducing the risk of spoilage (Ning et al., 2023). Nanoparticle-based systems have also been explored to improve endolysin stability and delivery. The chimeric endolysin Cpl-1, encapsulated within chitosan nanoparticles, demonstrated enhanced bioavailability and prolonged *in vivo* half-life during the treatment of *S. pneumoniae* infections (Gondil et al., 2020). Furthermore, in a mouse model of *S. aureus* osteomyelitis, an engineered endolysin incorporated into an alginate hydrogel exhibited improved stability and antibacterial activity while reducing cytotoxicity and bacterial burden. (Yao et al., 2021).

### 5.3 Addressing the outer membrane barrier of gram-negative bacteria using outer membrane permeabilizers

Many endolysins targeting Gram-negative bacteria exhibit limited antibacterial activity due to the presence of the outer membrane barrier, which restricts endolysin access to the underlying peptidoglycan layer. To overcome this limitation, several studies have investigated the use of chelators and outer membrane permeabilizers (OMPs), including trichloroethane, citric acid, Triton X-100, and ethylenediaminetetraacetic acid (EDTA). The combined application of endolysins with OMPs has demonstrated synergistic antibacterial activity against antibiotic-resistant Gram-negative bacteria (Khan et al., 2021). The outer membrane of Gram-negative bacteria consists of a phospholipid bilayer and lipopolysaccharides (LPS), which function as a protective permeability barrier. Treatment with OMPs may disrupt membrane integrity and increase membrane permeability, thereby facilitating endolysin access to peptidoglycan (Briers et al., 2011). Polycationic compounds such as poly-L-lysine, aminoglycosides, and polymyxins can interact with negatively charged LPS molecules and contribute to destabilization of the outer membrane structure (Ning et al., 2021). In addition, EDTA and citric acid have been used to enhance outer membrane permeability by disrupting membrane-associated ions and structural stability (Zhang et al., 2015). However, the application of EDTA may be limited because of concerns regarding its potential adverse effects on human cells. As an alternative approach, high hydrostatic pressure (HPP) has been investigated as a safer strategy for enhancing bacterial susceptibility. Gram-negative bacteria have shown increased susceptibility to bacteriocins and antimicrobial peptides when combined with HPP treatment (Bolumar et al., 2021).

### 5.4 Next-generation precision endolysins: bacterial receptor-mediated targeting

Enhancing endolysin specificity and facilitating penetration across the bacterial outer membrane represent important strategies for improving antibacterial efficacy against Gram-negative bacteria. One approach involves combining endolysins with bacteriocins to promote specific binding to bacterial cell surface receptors. Bacteriocins can interact with outer membrane phospholipids, receptors, and transport proteins, resulting in pore formation that may facilitate the transport of endolysins to the peptidoglycan layer of the bacterial cell wall (Martínez et al., 2016). *E. coli* and *Yersinia pestis* expressing the outer membrane transporter FyuA, which functions as a receptor for pesticin, were selectively targeted using the receptor-binding domain of pesticin conjugated with T4 lysozyme (Lukacik et al., 2012). Similarly, the combination of the endolysin LysEP3 with bacteriocin demonstrated antibacterial activity against targeted bacterial strain (Yan et al., 2017). Innolysins represent another strategy for overcoming the Gram-negative outer membrane barrier through the fusion of phage receptor-binding proteins (RBPs) with the enzymatically active domains (EADs) of endolysins (Zampara et al., 2020).

### 5.5 Artificial intelligence-driven design and high-throughput engineering

Artificial intelligence (AI) and machine learning are being explored for the identification of new endolysins from large phage genome datasets. Zhang et al. (2024) developed DeepLysin, an AI-based approach for identifying putative antibacterial lysins from uncharacterized phages. The authors selected 17 predicted lysins for experimental testing, and 7 of these showed antibacterial activity. One of the identified lysins, LLysSA9, showed activity against *S. aureus* and other tested Gram-positive bacteria (Zhang et al., 2024). The antibacterial activity of LLysSA9 was further examined using clinical *S. aureus* isolates. It was tested against 79 methicillin-susceptible *S. aureus* (MSSA) and 53 methicillin-resistant *S. aureus* (MRSA) isolates. LLysSA9 showed MIC_90_ values of 4 μg/mL for MSSA and 2 μg/mL for MRSA. Under the same experimental conditions, the natural phage lysin CF-301 showed MIC_90_ values of 16 μg/mL for both MSSA and MRSA. Thus, LLysSA9 showed lower MIC_90_ values than CF-301 in this study (Zhang et al., 2024). The authors also evaluated LLysSA9 in mouse models of *S. aureus* bloodstream and wound infections and reported antibacterial effects in both models (Zhang et al., 2024). High-throughput approaches have also been used for the engineering of endolysins. Gerstmans et al. (2020) developed the VersaTile platform for the modular construction and screening of engineered lysins. Using this platform, the authors generated a library of approximately 10,000 lysin variants and screened them for activity against *Acinetobacter baumannii*. The first library had an activity hit rate of about 2%, while a second library produced more than 21% active variants (Gerstmans et al., 2020). One selected variant, 1D10, reduced the bacterial count by up to 4.5 log units within 30 min in human serum. The variant was also tested in an *ex vivo* pig burn-wound model, where it prevented bacterial growth and biofilm development (Gerstmans et al., 2020). Recent work on endolysin engineering has described the use of computational approaches together with experimental screening. These approaches include sequence-based analysis, domain engineering, peptide fusion, and the construction of lysin libraries for experimental screening (Aitken et al., 2025). AI-assisted identification and high-throughput platforms such as VersaTile therefore provide different approaches for the discovery and engineering of endolysins.

As many reported Gram-negative endolysins exhibit reduced activity in complex biological media, such as serum and blood, these approaches may facilitate the development of novel engineered endolysins with improved stability and antibacterial activity, as illustrated in Figure 4.

**FIGURE 4:**
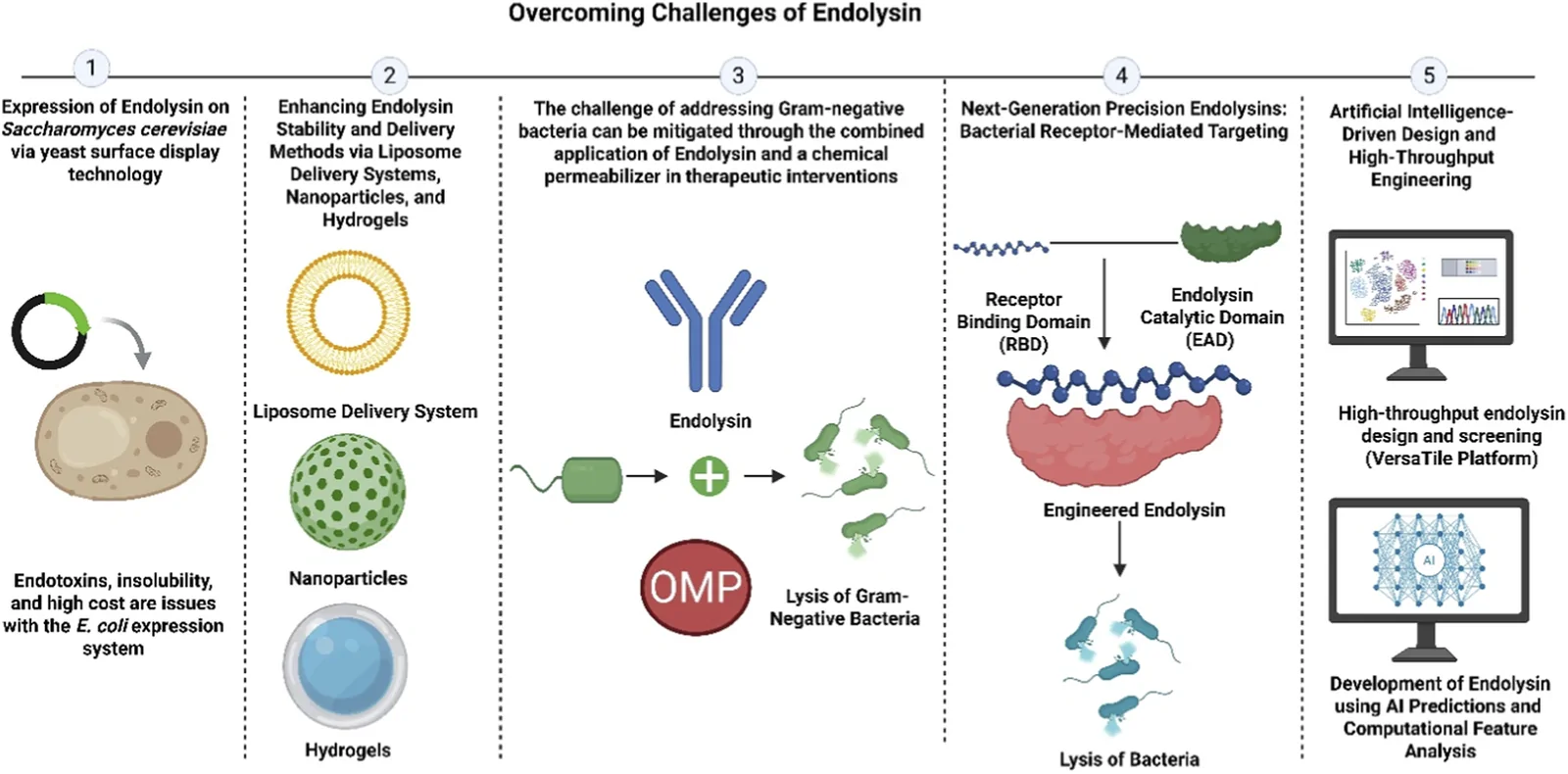
Illustrates the potential solutions for addressing the challenges of endolysins. Image was created by BioRender.com.

## 6 Conclusion

Bacteriophage-derived endolysins represent a promising class of alternative antimicrobial agents for combating multidrug-resistant (MDR) bacterial pathogens. Endolysins exert bactericidal activity by targeting and degrading the bacterial peptidoglycan layer with high specificity. In reported experimental systems, their targeted activity may reduce unintended effects on beneficial commensal microbiota compared with broad-spectrum antimicrobials. In addition to their therapeutic potential, the cell wall-binding domains (CBDs) of endolysins have been explored for the development of sensitive, specific, rapid, and cost-effective diagnostic probes as alternatives to conventional antibody-based detection methods.

Most reported endolysins exhibit stronger lytic activity against Gram-positive bacteria, whereas their activity against Gram-negative bacteria is often limited by the outer membrane barrier, which restricts access to the peptidoglycan layer. Various approaches have been investigated to overcome this limitation, including outer membrane permeabilizers, engineered lysins, receptor-mediated delivery systems, encapsulation technologies, and protein engineering strategies. Notably, engineering approaches can enhance the lytic activity of Gram-negative-targeting endolysins, enabling the rational design of more effective engineered variants specifically tailored against Gram-negative pathogens. This review also discusses important challenges associated with endolysin development, including difficulties in cloning and expression, reduced stability in complex biological media, and the high cost of large-scale production.

Emerging approaches, including artificial intelligence-assisted design, formulation and encapsulation strategies, alternative expression systems, and synthetic engineering platforms, may support the development of endolysins with improved stability, specificity, and antibacterial activity. Although several biological and translational challenges remain, endolysins continue to demonstrate considerable potential as antimicrobial, biocontrol, and diagnostic agents for addressing the growing threat of antimicrobial resistance (AMR).
